# Risk factors affecting chemical and bacteriological quality of bulk tank milk in Kerman, Iran

**Published:** 2015-03-15

**Authors:** Ladan Mansouri-Najand, Zeinab Rezaii

**Affiliations:** 1 *Department of Food Hygiene and Public Health, Faculty of Veterinary Medicine, Shahid Bahonar University of Kerman, Kerman, Iran;*; 2 *Graduate Student, Faculty of Veterinary Medicine, Shahid Bahonar University of Kerman, Iran.*

**Keywords:** Bulk tank milk, Milk quality, Risk factor

## Abstract

Milk is often described as a complete food because it contains protein, sugar, fat, vitamins, and minerals. This study was performed to investigate risk factors affecting chemical and bacteriological quality of bulk tank milk. According to the following conducted experiments, the milk was divided into two standard and non-standard groups. Then, effect of risk factors on making the samples non-standard was studied. Risk factors such as type of milk delivery unit, distance of cattle farm from plant, size of herd, education level of stockbreeders, capacity of milk transport tank, capacity of cooler device, and number of workers employed in cattle farms were evaluated in this study. Microbial and chemical evaluations were performed. Beta-lactam antibiotic residues and somatic cell count were specified. At the same time, the stockbreeders who referred to the plant were given some questionnaires and the mentioned primary questions were asked. After collecting the data, logistic regression model was used. According to the obtained results and comparison with Iran’s national standard, 26 out of 109 samples were determined to be at standard level and 83 ones had at least one out-of-standard factor. The results obtained from the model demonstrated significant effect of education of stockbreeders and capacity of cooler devices on the milk quality. Education of stockbreeders could greatly affect management of a cattle farm unit.

## Introduction

Importance of consuming milk and dairy products is undeniable for human nutrition.^1^ As a nutritional and balanced foodstuff, milk is a suitable medium for growth of several microorganism.^2^ Milk contains compounds required for the body; thus, its microbial and chemical health is one of the important issues which should be considered in dairy industries and communities, particularly in developing countries.^3^ The objective of this study was to evaluate microbiological and chemical quality of bulk tank milk in Kerman and determine risk factors affecting milk quality parameters.^4^

Quality of milk may be evaluated by measuring parameters that indicate its suitability for both consumption and processing into dairy products. The principal parameter which is routinely used in inter-national scale in this context is somatic cell count (SCC) and total bacterial count of milk.^5^

The SCC is an excellent indicator of subclinical mastitis. Somatic cells are mostly leukocytes and increase in milk almost entirely because of microbial infections.^6^

Antibiotic residues in milk are of great concern for dairy farmers, milk processors, regulatory agencies and consumers. In addition, effectiveness of antibiotics is threatened by extensive, inappropriate use. In veterinary practice, antibiotics are utilized at therapeutic levels primarily to treat diseases and to prevent infections. They are also used at sub-therapeutic levels to increase feed efficiency and promote growth in food producing animals. The frequent, sometimes illegal, use of anti-biotics may result in different concentration levels of residues which are found in products of animal origin, such as milk. Beta-lactams composes some of the antibiotics which are most frequently used for treatment of sick animals in Europe.^7^ Beta-lactams antibiotics (Penicillins and cephalosporins) are still the predominant drug residues in bulk tank milk.^8^

## Materials and Methods

Raw milk samples (n = 109) were collected in raw milk reception site of Milk Industries Plant. These samples were sent from dairy farms of Kerman, milk collecting centers and other plants to this site. The samples were sent to Food Hygiene Laboratory, Faculty of Veterinary Medicine, Shahid Bahonar University of Kerman, Kerman, Iran to perform total microbial count, psychrotroph and *Staphylococcus* *aureus* count tests. For counting aerobic mesophilic bacteria, nutrient agar and pure plate methods were used (Merck, Darmstadt, Germany). The plates were incubated at 32℃ ˚C for 72 hr. To count psychotropic bacteria, plate count agar (Merck, Darmstadt, Germany) was carried out at 30 ˚C for 48 hr. *Staphylococcus aureus* was cultured by surface plate method on Baird Parker agar (Merck, Darmstadt, Germany) and was incubated at 37 ˚C for 48 hr. The suspected colonies were checked by coagulase test.

Chemical content of bulk tank milk such as percent of protein, fat, lactose, solid not-fat and freezing point were measured by lactostar (Funke Gerber Inc., Berlin, Germany).

Residues of the microbial inhibitors were measured by Coupon Kit (Christian Hansen Company, Lyngby, Denmark). Also, residues of beta-lactam antibiotics were measured using beta star test (Neogen Co., Lansing, USA) and somatic cell counts were assessed by Fossomatic cell counter (Model FC 5000; Foss, Hillerod, Denmark). Electronic methods for counting somatic cells in milk were also described.^9^ It has been in use for both mastitis control and milk quality work since the method is rapid and cheap and has a reproducible result; it is non-subjective compared to other methods.^10^

In this regard, hygienic quality of bulk tank milk delivered to Kerman Milk Industries Plant was evaluated, compared with Iran’s national standards and assigned to standard and non-standard groups. Concurrent with collection of the samples, the questionnaires were given to stockbreeders. These questions were raised in the questionnaires: distance between dairy farm and site of plant (below 20 km, equal to or above 20 km), herd size (below 200 heads, equal to or above 200 heads), education of stockbreeders (above high school diploma, below high school diploma), capacity of milk tanks (above 1500 kg, equal to or below 1500 kg), capacity of cooler devices (above 1000 kg, equal to or below 1000 kg ), daily production (above 1000 kg, equal to or below 1000 kg) and number of workers in dairy farm (above five workers, equal to or below five worker). In order to analyze the factors affecting quality of milk delivered to Kerman Milk Industries Plant, logistic regression model was used. In this model, conformity with Iran’s national standards in all the performed tests and the raised questions were included as the dependent and independent variables, respectively. 

## Results

Based on the comparison with national standard criteria, 26 out of 109 samples were at standard levels in terms of all the factors and 83 samples were out of standard in terms of at least one of the factors (76.10%). Table 1 shows non-standard samples based on the performed tests and Table 2 shows the results obtained from the model. 

**Table 1:** Classification of the samples collected from Kerman milk industries plant based on standards of the country.

**Type of sample**	**Non-standard (%)**	**Confidence level (95%)**
**Total samples**	76.10	68.00-84.30
**Cattle farm**	73.70	64.70-82.70
**Collection center**	87.50	57.90-100
**Other plants**	100	0-0

**Table 2:** Crude odds ratio of nonstandard milk samples to education of stockbreeder, capacity of cooler and type of unit

**Variables**		**Crude odd ratio**	**Confidence level of 95% **	***p*** **-value**
**Education**	Above high school	1.00	-	-
	Below high school	4.10	1.10 - 15.3	0.03
**Capacity of cooler**	> 1000 L	1.00	-	-
	≤ 1000 L	2.90	1.05 - 7.90	0.04
**Type of units**	Cattle farm	1.00	-	-
	Collection centers	3.10	0.70 - 13.92	0.134

On this basis, quality of bulk tank milk in dairy farms with the owners who had below high school diploma was lower by 1.40% compared with the owners who held high school diploma and higher degrees (*p* < 0.03). Quality of bulk tank milk in the units which had small cooler devices (equal to or below 1000 kg) was lower than that of units with large ones (above 1000 kg) by 2.90% (*p* < 04). The results obtained from crude odds ratio also showed a relationship between type of unit and non-standard milk. However, it was not statistically significant in collection centers due to the small sample size. 

## Discussion

The results obtained from logistic regression model showed, that education of stockbreeders and capacity of coolers were effective in quality of the delivered milk. Education of the stockbreeders had a major role in management of an animal husbandry unit. Based on the results, the stockbreeders who held higher school degrees produced high-quality milk which could be due to better management of some issues such as use of new managerial programs, new devices, observance of hygiene and consideration of animal nutrition. Cooling of milk immediately after milking is vital for maintaining high-quality levels until being processed for consumption or use for manufacturing other dairy products. The results also showed that the dairy farms with cooler capacity of above 1000 liters produced milk with higher quality which could be attributed to preservation of milk at suitable temperature after milking. The milk produced in animal husbandry units had better quality than milk of other collection centers and surplus milk of other plants. Morlini *et al*. studied effect of milking tools and milking management on psychrotrophic count in Argentina and found that cooling milk in cold heat exchange plates had better effect on improvement of health quality than cold tanks.^11^ In the study which was conducted in Canada by Elmoslemany *et al*., it was found that use of fast coolers and observance of hygienic requirements were very effective in quality of milk at time of milking.^12^ In another study which was conducted by Elmoslemany *et al*., importance of udder health and milking system washing was regarded as the important risk factors in microbial quality of milk.^4^ In contrast with the present study, Jayarao *et al.* showed that herd size and farm management had considerable influence on somatic cell and bacterial counts in bulk tank milk.^13^ Elmoslemany *et al.* demonstrated increasing herd size as a risk factor.^12^

In conclusion, categorized bulk tank somatic cell and bacterial counts could serve as indicators and facilitate monitoring herd udder health and milk quality.^14^ In other studies conducted by Zucali *et al.* and Molineri *et al. *in 2011 and 2012, respectively, it was found a relationship with udder hygiene score. Routine Milking operation strongly affected bacterial counts and linear score of bulk tank milk.^11^^,^^15^ Bava *et al*. showed that cleaning temperature was related to psychrotrophic bacterial count of milk and post-rinse water and coliform count in liners. Routine checking and regulation of water temperature during the washing phase of the milking machine could be a simple and effective way for controlling one of the main risk factors for bacteriological quality of bulk tank milk.^16^ In another study which was conducted in 2003 by Ruegg *et al.,* bulk tank milk analysis was widely accepted as a useful tool for evaluating milk quality and monitoring udder health status.^17^ The bulk tank milk quality showed a strong relationship with the risk factors. 
